# 

**Published:** 1898-08

**Authors:** 


### International Dental Journal

JAMES TRUMAN, D.D.S., Editor.

GEORGE W. WARREN, D.D.S., Assistant Editor.

LIBRARY S.G.O.

VOL. XIX. AUGUST, 1898. No. 8.

PUBLISHED MONTHLY BY

THE INTERNATIONAL DENTAL PUBLICATION COMPANY,

716 FILBERT STREET, PHILADELPHIA.

EDITORIAL OFFICE, 4505 CHESTER AVENUE, PHILADELPHIA, PA.

$2.50 a Year, in Advance. Single Copies, 25 Cents.

Printed by J. B. Lippincott Company, Philadelphia.

Entered at the Post-Office at Philadelphia, and admitted for transmission through the mails at second-class rates.

